# Vertical Feedback Mechanism of Winter Arctic Amplification and Sea Ice Loss

**DOI:** 10.1038/s41598-018-38109-x

**Published:** 2019-02-04

**Authors:** Kwang-Yul Kim, Ji-Young Kim, Jinju Kim, Saerim Yeo, Hanna Na, Benjamin D. Hamlington, Robert R. Leben

**Affiliations:** 1 School of Earth and Environmental Sciences, Seoul National University, 1 Gwanak-ro, Gwanak-gu, Seoul, 08826 Republic of Korea; 2 APEC Climate Center 1463, Haeundae-gu, Busan, 48058 Republic of Korea; 3 Jet Propulsion Laboratory, California Institute of Technology, 4800 Oak Grove Drive, Pasadena, CA 91109 USA; 4 Colorado Center for Astrodynamics Research, Department of Aerospace Engineering Sciences, ECNT 320, 431 UCB, University of Colorado, Boulder, Colorado, 80309-0431 USA

## Abstract

Sea ice reduction is accelerating in the Barents and Kara Seas. Several mechanisms are proposed to explain the accelerated loss of Arctic sea ice, which remains to be controversial. In the present study, detailed physical mechanism of sea ice reduction in winter (December–February) is identified from the daily ERA interim reanalysis data. Downward longwave radiation is an essential element for sea ice reduction, but can primarily be sustained by excessive upward heat flux from the sea surface exposed to air in the region of sea ice loss. The increased turbulent heat flux is used to increase air temperature and specific humidity in the lower troposphere, which in turn increases downward longwave radiation. This feedback process is clearly observed in the Barents and Kara Seas in the reanalysis data. A quantitative assessment reveals that this feedback process is being amplified at the rate of ~8.9% every year during 1979–2016. Availability of excessive heat flux is necessary for the maintenance of this feedback process; a similar mechanism of sea ice loss is expected to take place over the sea-ice covered polar region, when sea ice is not fully recovered in winter.

## Introduction

Over the past decades, rapidly enhanced atmospheric warming has been observed in the Arctic^1–3^. The accelerated warming is pronounced in the lower troposphere during the cold season^4–6^. An accompanying drastic reduction of sea ice^7,8^ has pronounced implications for global climate changes by affecting energy exchange between ocean and atmosphere^9^, and is often referred to as a key factor for accelerated warming in the Arctic^10–12^. A particularly significant sea ice reduction can be found over the Barents and Kara Seas, which potentially influences cold winter extremes over the Eurasian continent^13–19^. Physically, sea ice loss involves a positive ice-atmosphere feedback, which leads to an enhanced warming signal in the Arctic region. This feature is generally referred to as Arctic amplification^6,9,20^. Previous studies have proposed the physical mechanisms of Arctic amplification, which involve the effect of atmospheric heat transport^21,22^, oceanic heat transport^23–26^, cloud and water vapor changes^27–32^, and/or diminishing sea ice cover^5,6,33^. The accurate physical process of the Arctic amplification, however, is subject to debate.

Due to the large seasonal variation of insolation, there exists pronounced seasonality in the air-sea interaction process over the Arctic Ocean. During summer, open water readily absorbs solar radiation, which results in increasing heat content in the oceanic mixed layer. This represents the so-called albedo feedback^5,6,9,34,35^, meaning that the Arctic Ocean is efficient in absorbing atmospheric heat during summer. The albedo feedback is also important during the snow and ice melt in spring and early summer even before the appearance of open sea. After the sun sets over the Arctic Ocean, the ice-albedo feedback is suppressed and the primary air-sea interaction mechanism becomes oceanic horizontal advection and vertical convection of heat^36^. The stored heat in the ocean mixed layer is released back to the colder atmosphere above, which will result in warming of the atmosphere. The decreased insulation effect^36^ due to the loss of sea ice also promotes further sea ice reduction. Thus, heat transfer between the ocean and atmosphere is generally considered as the fundamental mechanism of Arctic amplification, which is pronounced only during the cold season. On the other hand, increased cloud cover and water vapor^27–32,37^ can also contribute to an increase in downward longwave radiation.

Despite the general consensus that heat transfer between the ocean and atmosphere is a crucial element in the physical mechanism of Arctic amplification and sea ice reduction, a quantitative understanding of individual contributions of heat flux components is still controversial. Further, the role of upward and downward longwave radiation in Arctic amplification is vague and not fully understood. Accurately quantifying the contribution of these different mechanisms, therefore, is required for a complete understanding of the Arctic amplification.

In the previous study^33^, we showed that the temporal pattern of sea ice variation indeed differs significantly between the Barents–Kara Seas and the Laptev and Chukchi Seas. Sea ice refreezes and the sea surface exposed to air is closed up in late fall in the Laptev and Chukchi Seas. As a result, significant absorption of solar radiation in summer does not lead to increased turbulent heat flux in winter. However, sea surface does not freeze up completely in the Barents–Kara Seas. Consequently, we hypothesis that turbulent heat flux becomes available in winter in the Barents–Kara Seas for heating the atmospheric column, which in turn increases downward longwave radiation.

In the present study, a quantitative assessment of energy fluxes involved in the Arctic amplification is investigated in relation to the sea ice reduction over the Barents and Kara Seas. This is an extension of the previous study with a specific goal of delineating the feedback mechanism between sea surface and the atmosphere. In particular, we extract a physically meaningful warming signal in the Arctic region and investigate how sea ice loss and individual energy fluxes are linked in a quantitative manner. For this goal, cyclostationary empirical orthogonal function (CSEOF) analysis^38–40^ is carried out on surface and pressure-level variables derived from the ERA interim daily reanalysis data^41^ in winter (Dec. 1–Feb. 28, *d* = 90 days). It should be noted that our discussion is restricted to processes in the Arctic; forcing from lower latitudes can also be important in the process of Arctic amplification and sea ice reduction.

## Results and Discussion

**Figure 1:**
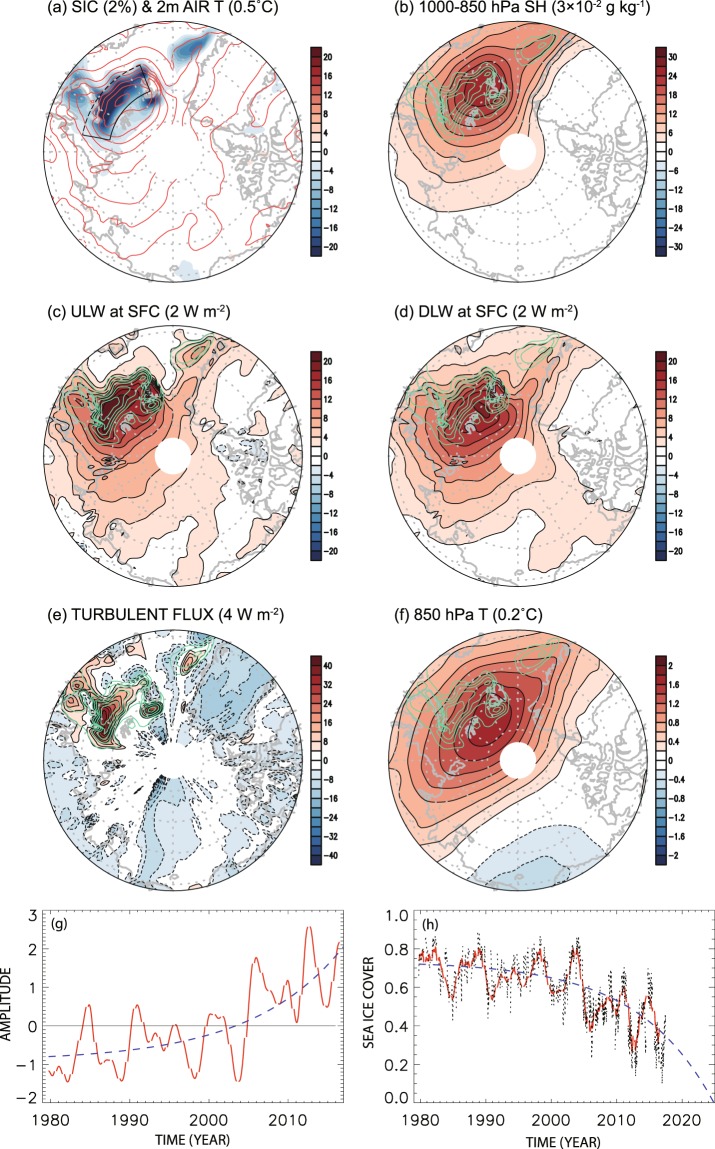
Winter (Dec. 1–Feb. 28) average patterns of sea ice loss mode: (**a**) sea ice (*shading*) and 2 m air temperature (*contour*), (**b**) 1000–850 hPa specific humidity, (**c**) upward longwave radiation, (**d**) downward longwave radiation, (**e**) turbulent (sensible + latent) heat flux, (**f**) 850 hPa air temperature, (**g**) the corresponding amplitude change (*red solid curve*) and the amplification curve (*blue dashed curve*), and (**h**) actual sea ice change in the sea-ice loss region (21°–79.5°E × 75°–79.5°N; the boxed area in (a)) of the Barents and Kara Seas (*black dotted curve*; extended until 2017 based on new data), sea ice change according to the sea ice loss mode (*red curve*), projection based on the amplification curve (*blue dashed curve*). The red curve in (**h**) is obtained by multiplying the loading vector of sea ice concentration (**a**) averaged in the boxed area with the amplitude time series (**g**) according to equation (7). The green contours in (**b**–**f**) represent sea ice concentration in (**a**). The numbers in parenthesis are contour intervals and negative contours are dashed. Figures in (**a**–**f**) were created with GrADS 2.1.0 (http://cola.gmu.edu/grads/).

Figure 1 shows the sea ice loss mode identified through CSEOF analysis. Since the loading vector (Fig. 1a; see also Figs S1 and S2 in the supplementary information) and the amplitude (PC) time series (Fig. 1g) describes the sea ice reduction, together with natural variability of sea ice concentration, this mode represents the loss of sea ice in the Barents and Kara Seas during the past 37 years and explains 24% of the total variability of the sea ice concentration in the Arctic Ocean. The pattern of sea ice reduction (Fig. 1a) is nearly identical with the trend pattern of sea ice concentration in the Arctic Ocean (see Fig. S1). As can be seen in Fig. 1h, the sea ice reduction trend in the Barents and Kara Seas (boxed area in Fig. 1a) is captured by this mode. In particular, the rate of sea ice loss has significantly increased since 2004–2005^42^. In association with the sea ice loss, 2 m air temperature, 850 hPa temperature, specific humidity, upward longwave radiation, downward longwave radiation, and upward heat flux have increased significantly over the region of major sea ice loss [21°–79.5°E × 75°–79.5°N] (boxed area in Fig. 1a). Multiplying the amplitude time series (Fig. 1g) with the loading vector (Fig. S2) of the sea ice loss mode as in equation (7), actual sea ice concentration time series is obtained as in Fig. 1h. According to Fig. 1h, sea ice concentration has decreased by ~40% during the last 37 years (1979–2016).

As can be seen in Fig. 1a,c and e, the central areas of anomalous 2 m air temperature, upward longwave radiation and turbulent (sensible + latent) heat flux match well with the region of sea ice loss^36^. On the other hand, the centers of the downward longwave radiation and lower-tropospheric specific humidity match well with that of the 850 hPa air temperature (Fig. 1b,d and f).

**Figure 2:**
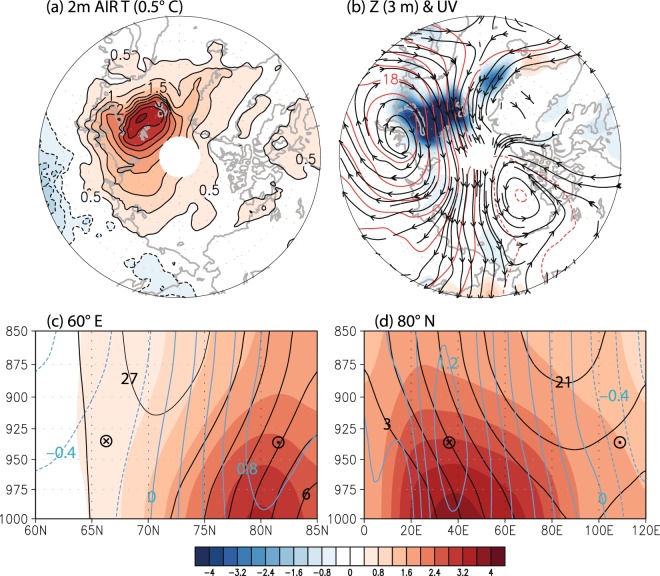
Winter-averaged patterns of anomalous atmospheric condition: (**a**) 2 m air temperature (0.5 °C contour interval), (**b**) lower tropospheric (1000–900 hPa) geopotential height (*red contour*; 3 m contour interval) and wind (*black contour*), sea ice reduction (%, *shading*), (**c**) vertical cross section along 60 °E of lower tropospheric (1000–850 hPa) air temperature, geopotential height and wind, and (**d**) along 80 °N. Temperature is in shading (0.4 K contour interval), geopotential height is in black contours (3 m contour interval), and (**c**) zonal and (**d**) meridional winds are in blue contours (0.2 m s^−1^ contour interval). Figures were created with GrADS 2.1.0 (http://cola.gmu.edu/grads/).

Figure 2 shows the anomalous surface (2 m) air temperature, the lower tropospheric geopotential height and wind and the vertical cross section of anomalous temperature, geopotential height and wind along 60°E and 80°N associated with the sea ice reduction. A significant warming is seen in the lower troposphere^3,4,12^. Note that the anomalous temperature pattern is similar to the second EOF pattern in Graversen *et al*.^21^. The anomalous temperature and geopotential height are consistent according to the hydrostatic equation (see Fig. S3). Anomalous wind and geopotential height are consistent according to the thermal wind equation. As can be seen, an anticyclonic circulation is established over the region of sea ice loss. This anticyclonic circulation results in advection of warmer air over the Barents and Kara Seas and advection of colder air over the mid-latitude East Asia^19^.

The winter-averaged patterns of anomalous downward longwave radiation and specific humidity look fairly similar to that of 850 hPa air temperature (Figs 1 and S4). It appears that the increased downward longwave radiation is the result of the tropospheric warming (Fig. 2). Specific humidity also increases with the tropospheric warming. Note specifically that these changes are observed over or close to the region of sea ice reduction. The pattern of total cloud cover, however, differs significantly from that of sea ice reduction. Since cloud is a difficult variable to simulate accurately, we also examine total column liquid water and total column ice water, which are the key variables for the formation of clouds. The patterns of total column liquid water and total column ice water exhibit a strong response over the region of sea ice reduction although their centers of action are shifted toward the Greenland Sea (Fig. S4d). Therefore, we postulate that the increased downward longwave radiation is due to the increased 850 hPa air temperature and the greenhouse effect produced by the increased specific humidity and cloudiness to a lesser extent; this is consistent with several previous studies^43,44^. Further note that net (upward minus downward) longwave radiation is positive over the region of major sea ice reduction, whereas it is slightly negative over the surrounding areas (Fig. S4c). Thus, at the surface level, there is a net loss of longwave energy over the region of sea ice reduction, while there is a net gain of longwave radiation over the surrounding area.

**Figure 3:**
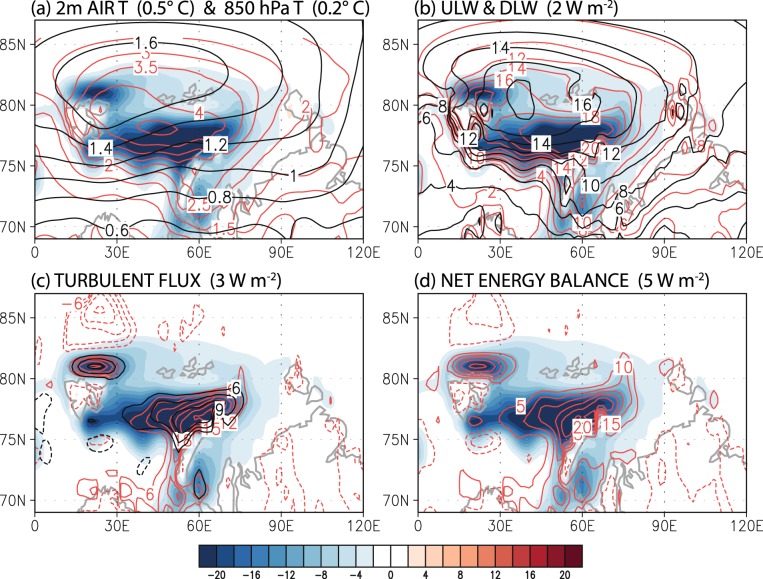
Winter average pattern of sea ice loss mode in the Barents and Kara Seas: (**a**) sea ice reduction (%, *shading*), 2 m air temperature (*red contour*) and 850 hPa temperature (*black contour*), (**b**) upward longwave radiation (*red contour*) and downward longwave radiation (*black contour*), (**c**) sensible heat flux (*red contour*) and latent heat flux (*black contour*), and (**d**) net energy balance (sensible heat flux + latent heat flux + upward longwave radiation – downward longwave radiation). Figures were created with GrADS 2.1.0 (http://cola.gmu.edu/grads/).

**Figure 4:**
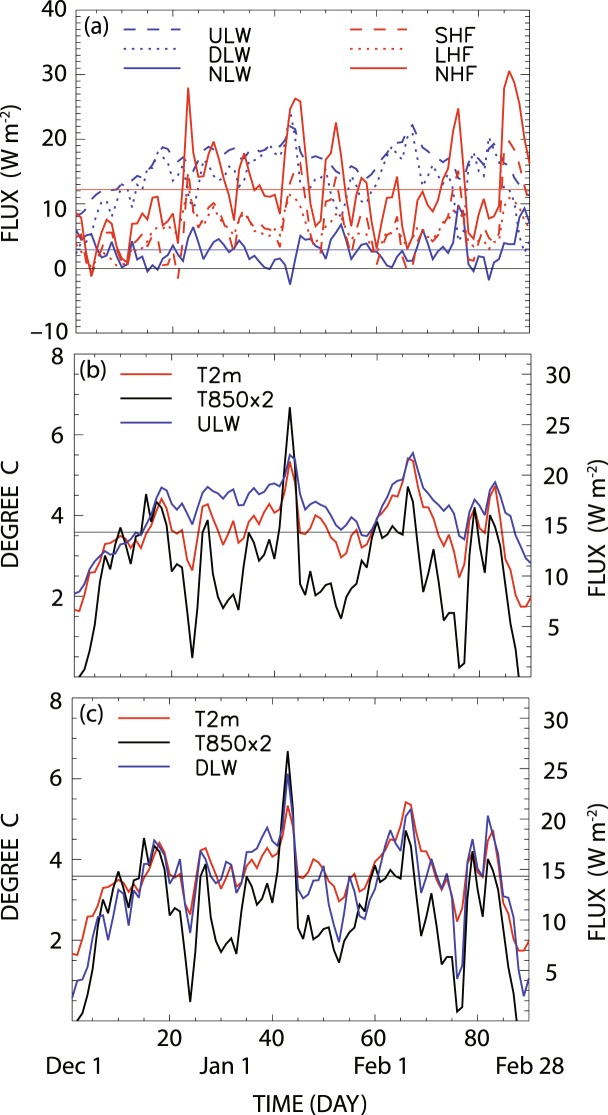
Daily patterns of variability over the region of sea ice loss (21°–79.5°E × 75°–79.5°N): (**a**) upward longwave radiation (*blue dashed*), downward longwave radiation (*blue dotted*), net longwave radiation (*blue solid*) with its mean value (*blue straight line*), sensible heat flux (*red dashed*), latent heat flux (*red dotted*), and turbulent heat flux (*red solid*) with its mean value (*red straight line*), (**b**) 2 m air temperature (*red*), 850 hPa air temperature × 2 (*black*), and upward longwave radiation (*blue*), and (**c**) same as (**b**) except for the regressed downward longwave radiation (*blue*). The straight lines in (**b**) and (**c**) represent the winter mean value of anomalous 2 m air temperature. Correlation of upward and downward longwave radiation with 2 m air temperature is respectively 0.88 and 0.91, whereas with 850 hPa air temperature is 0.66 and 0.85. Winter days are counted from December 1.

A prominent source of energy available for heating the atmospheric column is the increased turbulent heat flux from the wider area of sea surface exposed to air due to sea ice reduction (Fig. 3). Figure 4 shows the winter daily variations of the regressed loading vectors in equation (12) (terms in curly braces) averaged over the region of sea ice reduction (21°–79.5°E × 75°–79.5°N); it may be interpreted as the atmospheric response to the sea ice reduction shown in Fig. S2. Although the total (area-weighted) magnitudes of sensible and latent heat fluxes are generally smaller than those of upward and downward longwave radiation (Fig. 4a), turbulent heat flux is locally more pronounced than longwave radiation (Fig. 3)^35^. Furthermore, the combined effect of turbulent heat flux is about 6 times larger than that of longwave radiation, since upward and downward longwave radiation tends to offset each other and the resulting net longwave radiation is comparatively smaller than the net upward turbulent heat flux (Fig. 4a). In the presence of turbulent heat flux, air temperature and, henceforth, downward longwave radiation can increase continually leading to further sea ice reduction.

While the increased downward longwave radiation is a key element of sea ice reduction, it is not a sustainable physical process by itself. The area-averaged magnitudes of the upward and downward longwave radiation exceed those of the sensible and latent heat flux in the Barents and Kara Seas (Fig. 4a). The net amount of upward longwave radiation, however, is much smaller than the net upward heat flux as a result of near cancellation between the upward and downward longwave radiation. In fact, the upward radiation is, in general, slightly larger than the downward radiation resulting in a net upward longwave radiation of ~2 W m^−2^ in winter in the Barents and Kara Seas. This implies that surface temperature should decrease. A decrease in surface air temperature also means that upward longwave radiation decreases and, as a result, tropospheric air temperature decreases as well. In this sense, longwave radiation alone is not sufficient to sustain the sea ice reduction process. On the other hand, the net amount of heat flux is ~12 W m^−2^ in the same area. Once ocean surface is exposed due to the reduction of sea ice by ocean current^45,46^ or wind^30^, the enhanced turbulent heat flux helps sustain sea ice reduction by increasing downward longwave radiation. However, the release of turbulent heat flux can continue only when sea surface remains open. While an accurate energy budget is difficult to evaluate in the context of data analysis, Fig. 1a and g indicate that open sea surface area tends to increase in time, leading to increasing turbulent heat flux from the surface in the Barents-Kara Seas (see also Fig. 1e). This indicates that sea ice concentration is not fully recovered every year and turbulent heat flux increases as open sea surface area expands. Heat transport by the warm Norwegian current may be a likely mechanism for keeping the sea surface from freezing^23,26,45,46^.

As can be seen in Fig. 4b and c, daily upward longwave radiation change over the sea ice loss region is highly correlated with the daily fluctuation of 2 m air temperature, whereas daily downward longwave radiation change is strongly correlated with both 850 hPa and 2 m air temperatures. According to the lagged correlations (Fig. S5), daily changes of both upward and downward longwave radiation in the sea ice loss mode are highly correlated with those of 2 m air temperature and 850 hPa air temperature to a lesser extent. According to analysis based on 3-hourly data, 850 hPa air temperature leads changes in downward longwave radiation. Change in 2 m air temperature, on the other hand, is nearly simultaneous with the downward longwave radiation, whereas it slightly leads the upward longwave radiation. It appears that the increased tropospheric temperature increases the downward longwave radiation, which leads to a sea ice reduction. As a result, surface temperature and upward longwave radiation may increase.

**Figure 5:**
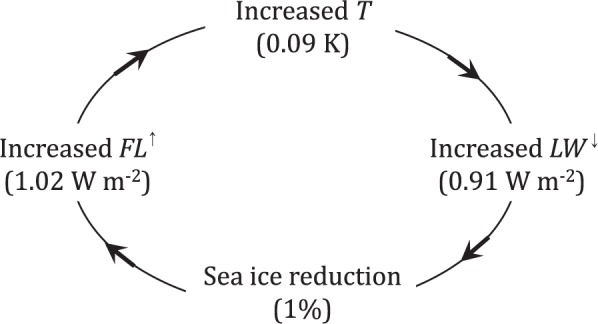
A proposed mechanism of polar amplification. Increased net upward energy flux increases air temperature. As a result, downward longwave radiation increases, which results in sea ice reduction. This loop seems to amplify by ~8.9% annually.

Therefore, we propose a feedback mechanism as suggested in Fig. 5. Sea ice reduction in this area leads to an increase in upward heat flux, which is used to raise temperature in the lower troposphere. Warming in the lower troposphere increases downward longwave radiation. As a result, sea ice reduction is accelerated. This feedback process can be written mathematically as follow:Step 1:

1 $$\frac{dF{L}^{\uparrow }}{dt}=-\,\alpha \frac{dS}{dt},F{L}^{\uparrow }=S{W}^{\uparrow }-\,S{W}^{\downarrow }+L{W}^{\uparrow }-\,L{W}^{\downarrow }+S{F}^{\uparrow }+L{F}^{\uparrow },$$

Step 2:

2 $$\frac{dT}{dt}=\beta \frac{dF{L}^{\uparrow }}{dt},$$

Step 3:

3 $$\frac{dL{W}^{\downarrow }}{dt}=\gamma \frac{dT}{dt},$$

Step 4:

4 $$\frac{dS}{dt}=-\,\delta \frac{dL{W}^{\downarrow }}{dt},$$

where *S* is sea ice concentration, *T* is tropospheric (850 hPa) temperature, *LW*^↓^ is downward longwave radiation, and the net upward flux *FL*^↑^ is the sum of net short and longwave radiation and sensible and latent heat fluxes. According to the winter (90-day) averaged loading vector of the sea ice loss mode, *α* = 1.016 × 10^2^, *β* = 9.522 × 10^−2^, *γ* = 1.155 × 10^1^, and *δ* = 8.946 × 10^−3^. It is emphasized that sea ice reduction continues, since downward longwave radiation continues to increase via enhanced upward heat flux from the exposed sea surface. According to our model, 1% reduction in sea ice coverage leads to 1.02 W m^−2^ increase in upward energy flux, which, in turn, leads to 0.09 K increase in 850 hPa air temperature and 0.91 W m^−2^ increase in downward longwave radiation. This process is being amplified according to the amplitude time series in Fig. 1g. As sea ice concentration dwindles as in Fig. 1h, turbulent heat flux and upward longwave radiation increase and, as a result, the lower tropospheric temperature and downward longwave radiation increase. It should be noted that net surface longwave radiation is upward so that surface cools via this mechanism. However, surface cooling is slower because of the increased downward longwave radiation, which delays sea ice freeze-up—key to this feedback loop.

This proposed feedback mechanism, in its present form, does not require any delayed action of increased absorption of insolation during summer in terms of albedo feedback. In winter, a significant amount of turbulent heat flux can be released from the ocean exposed to cold air without excessive energy stored in summer. Summer heating, on the other hand, may be a fortifying factor for this feedback loop by preventing sea ice from refreezing during fall and winter.

It should be noted that there are other processes, particularly forcing from lower latitudes, which are important for Arctic amplification and sea ice reduction. As can be seen in Fig. S6a and b, there are net convergence of moisture transport and heat transport over the region of sea ice reduction, although the center of action is over the Greenland Sea. Thus, moisture and heat transports from lower latitudes apparently affect the variation of sea ice concentration^43,44^. On the other hand, the horizontal transports of moisture and heat cannot explain one essential element of specific humidity anomaly and air temperature anomaly, respectively. As can be seen in Fig. S6c and d, moisture and heat transports contribute only about 30–40% of the mean value of anomalous specific humidity and air temperature, respectively. The remainder should derive from a vertical process. Therefore, vertical processes are an important mechanism for explaining winter sea ice reduction^47^.

According to the amplitude time series in Fig. 1g, the rate of sea ice reduction appears to be accelerating. A curve fit with an exponential function results in

5 $$pc(t)=a\exp (\lambda t)+b=a{({e}^{\lambda })}^{t}+b\approx a{(1+\lambda )}^{t}+b,$$

where *pc*(*t*) is the amplitude time series in Fig. 1g, and *t* is time in years since 1979. We obtained the fitting curve (dashed curve in Fig. 1g) with parameters *a* = 1.275 × 10^−1^, *λ* = 8.916 × 10^−2^, and *b* = −9.055 × 10^−1^. Equation (5) can be rewritten as

6 $$pc(t)-c=(pc(0)-c){(1+\lambda )}^{t}.$$

That is, the amplitude of sea ice reduction and atmospheric warming increases at the rate of ~8.9% every year.

## Methods

### Data

ECMWF Reanalysis (ERA) interim daily variables are used from 1979–2016^41^. Both surface and pressure-level variables during winter (Dec. 1–Feb. 28) are analyzed over the Arctic region (north of 60° N) to understand the detailed physical mechanism of sea ice loss and Arctic amplification.

### CSEOF analysis and regression analysis in CSEOF space

Analysis tool used for this study is the CSEOF technique^38–40^. In CSEOF analysis individual physical processes in space-time data are decomposed as:

7 $$T(r,t)=\sum _{n}{B}_{n}(r,t){T}_{n}(t),\,{B}_{n}(r,t)={B}_{n}(r,t+d),$$

where *B*_*n*_(*r*, *t*) depicts daily winter evolution of the *n*th physical process and *T*_*n*_(*t*) describes how the amplitude of the evolution varies on a longer time scale, and *r* and *t* denote location and time, respectively. Since the nested period *d* = 90 days, each loading vector, *B*_*n*_(*r*, *t*), consists of 90 spatial patterns which depict evolution of a variable throughout the winter. These winter evolution patterns, *B*_*n*_(*r*, *t*), repeat every winter, but its amplitude varies from one year to another according to the corresponding PC time series. CSEOF loading vectors are mutually orthogonal to each other in space and time and represent distinct physical processes. The principal component (PC) time series, *T*_*n*_(*t*) are uncorrelated with (and are often nearly independent of) each other. Each loading vector depicts a temporal evolution of spatial patterns seen in a physical process (such as El Niño or seasonal cycle), and corresponding PC time series describes a long-term modulation of the amplitude of the physical process. Thus, the CSEOF technique is suitable for extracting and depicting temporal evolution of (nearly independent) physical processes and often yields valuable insight that cannot be attained from single spatial pattern.

In order to make suitable physical interpretation of the analysis results, CSEOF analysis is conducted on a number of key variables. It is, then, extremely important to make CSEOF loading vectors derived from individual variables to be physically consistent with each other. For the purpose of generating physically consistent CSEOF loading vectors, regression analysis is carried out in CSEOF space^40^. A target variable is chosen such that its major CSEOF mode best depicts the physical process under investigation; target variable is sea ice concentration in the present study.

Once CSEOF analysis on the “target” variable is completed as in equation (7), physically consistent loading vectors of another variable, called the “predictor” variable, are obtained as follows:

Step 1: CSEOF analysis on a new variable

8 $$P(r,t)=\sum _{n}{C}_{n}(r,t){P}_{n}(t)$$

Step 2: regression analysis on a target PC time series

9 $${T}_{n}(t)=\,\sum _{m=1}^{M}{\alpha }_{m}^{(n)}{P}_{m}(t)$$

Step 3: construction of regressed loading vector

10 $${Z}_{n}(r,t)=\,\sum _{m=1}^{M}{\alpha }_{m}^{(n)}{C}_{m}(r,t)$$

Then, the target and predictor variables together can be written as

11 $$\{T(r,t),\,P(r,t)\}=\sum _{n}\{{B}_{n}(r,t),\,{Z}_{n}(r,t)\}{T}_{n}(t).$$

Namely, the loading vectors of the two variables, *B*_*n*_(*r*, *t*) and *Z*_*n*_(*r*, *t*), share an identical PC time series, *T*_*n*_(*t*), for each mode *n*. As a result, the evolution of a physical process manifested as *B*_*n*_(*r*, *t*) and *Z*_*n*_(*r*, *t*) in two different variables is governed by a single amplitude time series. Otherwise, *B*_*n*_(*r*, *t*) and *Z*_*n*_(*r*, *t*) do not represent the same physical process and henceforth are not physically consistent. This process can be repeated for as many predictor variables as needed. As a result of regression, then, entire data can be written in the form

12 $$Data(r,t)=\sum _{n}\{{B}_{n}(r,t),{Z}_{n}(r,t),{U}_{n}(r,t),\ldots \}{T}_{n}(t),$$

where the terms in curly braces denote physically consistent evolutions derived from various physical variables. A rigorous mathematical explanation of the regression analysis in CSEOF space can be found in Kim^48^.

## Supplementary information

Supplementary Information
