# Supplementary material for: Vertical Feedback Mechanism of Winter Arctic Amplification and Sea Ice Loss
Source: Sci Rep. 2019 Feb 4;9:1184. doi: 10.1038/s41598-018-38109-x (PMC6362226; doi:10.1038/s41598-018-38109-x)
Supplement: Supplementary file 1 — Supplementary Information [file 41598_2018_38109_MOESM1_ESM.pdf]

Supplementary Information

Vertical Feedback Mechanism of Winter Arctic Amplification and Sea Ice Loss

Kwang-Yul Kim[1*], Ji-Young Kim[1], Jinju Kim[1], Saerim Yeo[2], Hanna Na[1], Benjamin D. Hamlington[3], and Robert R. Leben[4]

[1]School of Earth and Environmental Sciences, Seoul National University, 1 Gwanak-ro, Gwanak-gu, Seoul 08826, Republic of Korea

[2]APEC Climate Center 1463, Haeundae-gu, Busan 48058, Republic of Korea

[3]Department of Ocean, Earth and Atmospheric Sciences, 4600 Elkhorn Avenue, Room 406, Old Dominion University, Norfolk, Virginia 23529, USA

[4]Colorado Center for Astrodynamics Research, Department of Aerospace Engineering Sciences, ECNT 320, 431 UCB, University of Colorado, Boulder, Colorado 80309-0431, USA

**Correspondence to*: Kwang-Yul Kim (kwang56@snu.ac.kr)

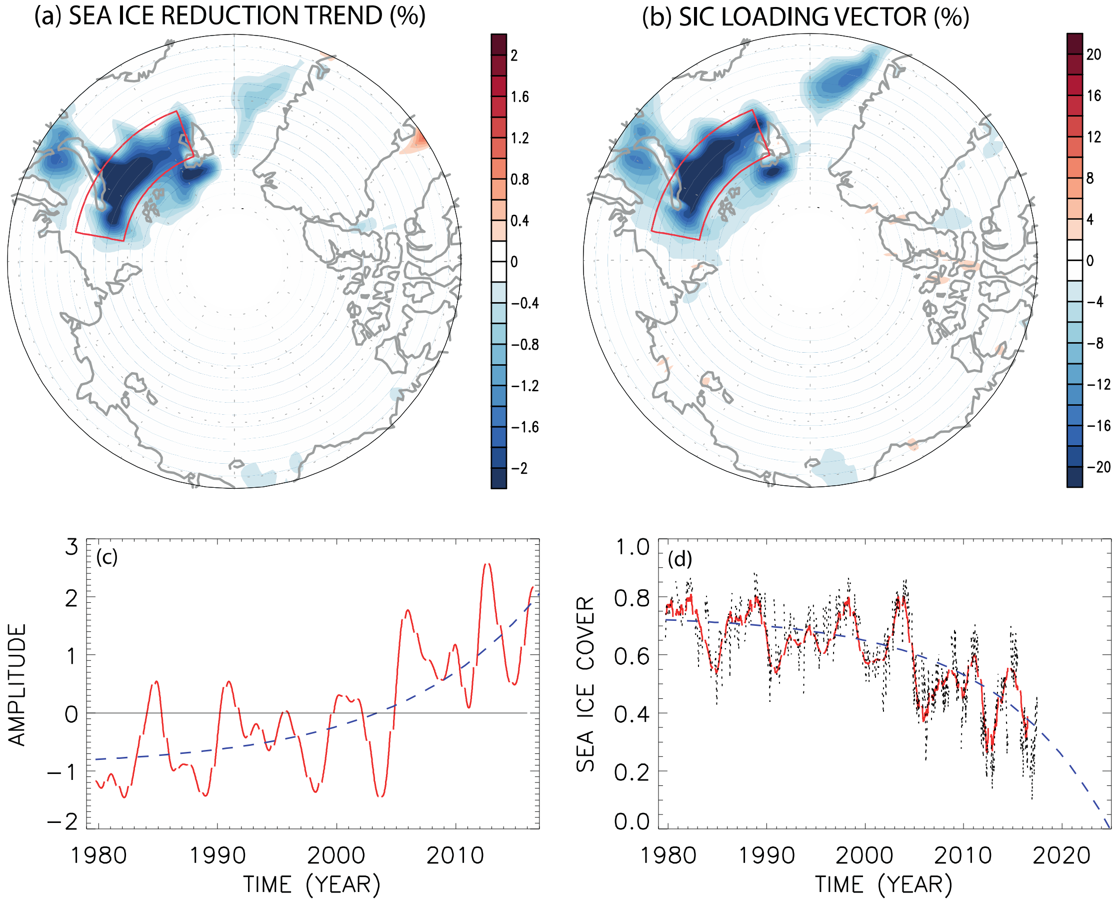

Figure S1. (a) The yearly trend (%) of sea ice reduction in the Arctic Ocean during 1979–2017, (b) the winter averaged loading vector of the sea ice loss mode, (c) the corresponding PC (amplitude) time series, and (d) actual sea ice concentration in the boxed area (black curve), sea ice concentration according to the sea ice loss mode (red curve) and a projection (blue dashed curve) based on the exponential fit of the amplitude time series in (c). Figures in (a)–(b) were created with GrADS 2.1.0 (http://cola.gmu.edu/grads/).

Figure S1a is obtained based on a linear trend of sea ice concentration at each grid point based on the ERA-Interim sea ice concentration from 1979–2017. This pattern is nearly identical with the sea ice loss mode discussed in the main text (Fig. S1b). Sea ice reduction is most conspicuous in the Barents-Kara Seas. The amount of sea ice reduction based on the sea ice loss mode is obtained by multiplying the loading vector (Fig. S1b) with its amplitude time series (Fig. S1c), resulting in Fig. S1d. As can be seen in Fig. S1d, the sea ice concentration change due to the sea ice loss mode (red curve) is similar to the actual data (black curve) with a fairly similar rate of trend.

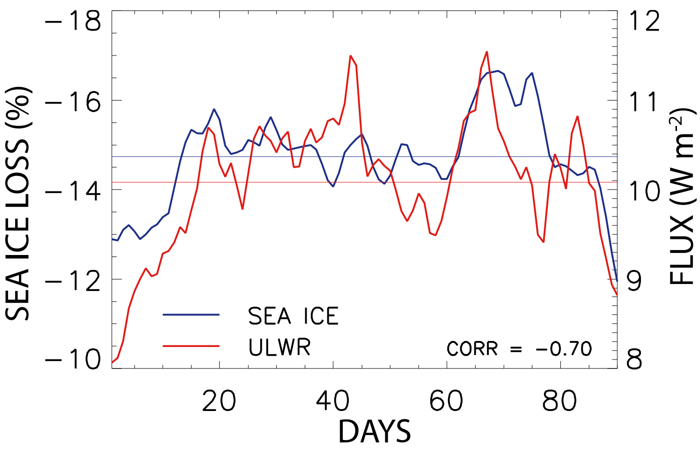

Figure S2. Anomalous daily sea ice concentration (*blue*) and upward longwave radiation (*red*) averaged over the region of sea ice loss (21°–79.5° E × 75°–79.5° N) with respective mean values (*straight lines*). Winter days are counted from December 1.

Sea ice concentration varies slightly on a daily basis, and its fluctuation is less than 2% from the mean value of −14.7% throughout the winter (Fig. S2). In accordance with the reduced sea ice concentration, upward longwave radiation flux is increased from the warmer sea surface exposed to air.

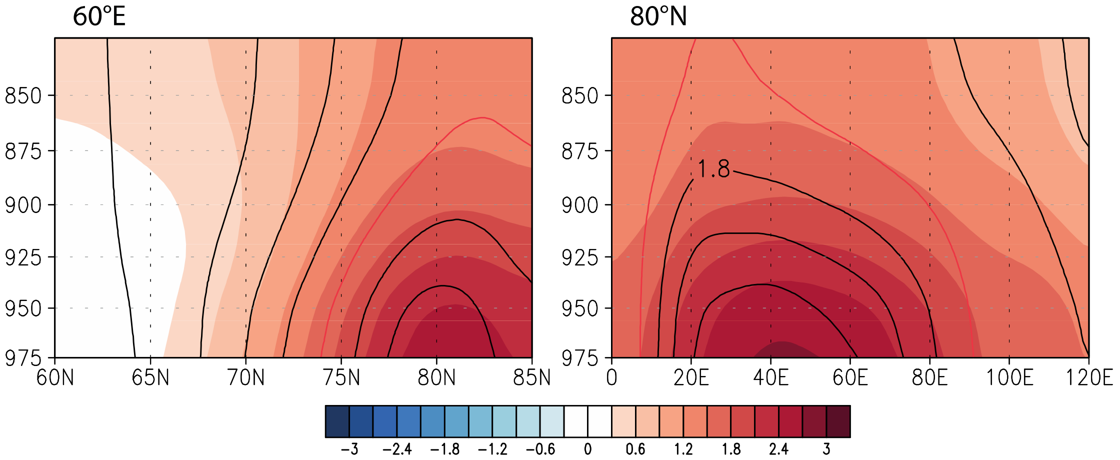

Figure S3. The pressure layer thickness ($\Delta Z = Z(p_1) - Z(p_0)$) derived from the geopotential height pattern in Fig. 2 in the text (shading) and that derived from the hydrostatic equation (contour). The red contour represents the thickness of 1.5 m. The level p_1 is the level used for plotting and p_0 is the pressure level below p_1 at the interval of 25 hPa.

The shaded geopotential height anomaly in this figure is obtained directly from the geopotential height field in Fig. 2 in the main text, i.e.,

$$(dZ)_j = Z_j - Z_{j-1}, \qquad (1)$$

where j is an index for the vertical level. The contoured geopotential height anomaly is obtained from the temperature field in Fig. 2 in the text, i.e.,

$$(dZ)_j = -\frac{R\langle T\rangle_j}{g}(d\ln p)_j, \qquad (2)$$

where

$$(dZ)_j = Z_j - Z_{j-1}, \quad \langle T\rangle_j = \left(T_j + T_{j-1}\right)/2, \quad (d\ln p)_j = \ln p_j - \ln p_{j-1}. \qquad (3)$$

As can be seen in Fig. S3, the anomalous geopotential height field is nearly in hydrostatic balance with the anomalous temperature field. The difference is partially due the use of layer mean temperature $\langle T\rangle$ in a finite-difference approximation of the hydrostatic equation in (2). Thus, it seems that the release of energy in the form of radiation and heat flux changes the temperature, and geopotential height in the lower troposphere adjusts in accordance with the hydrostatic balance.

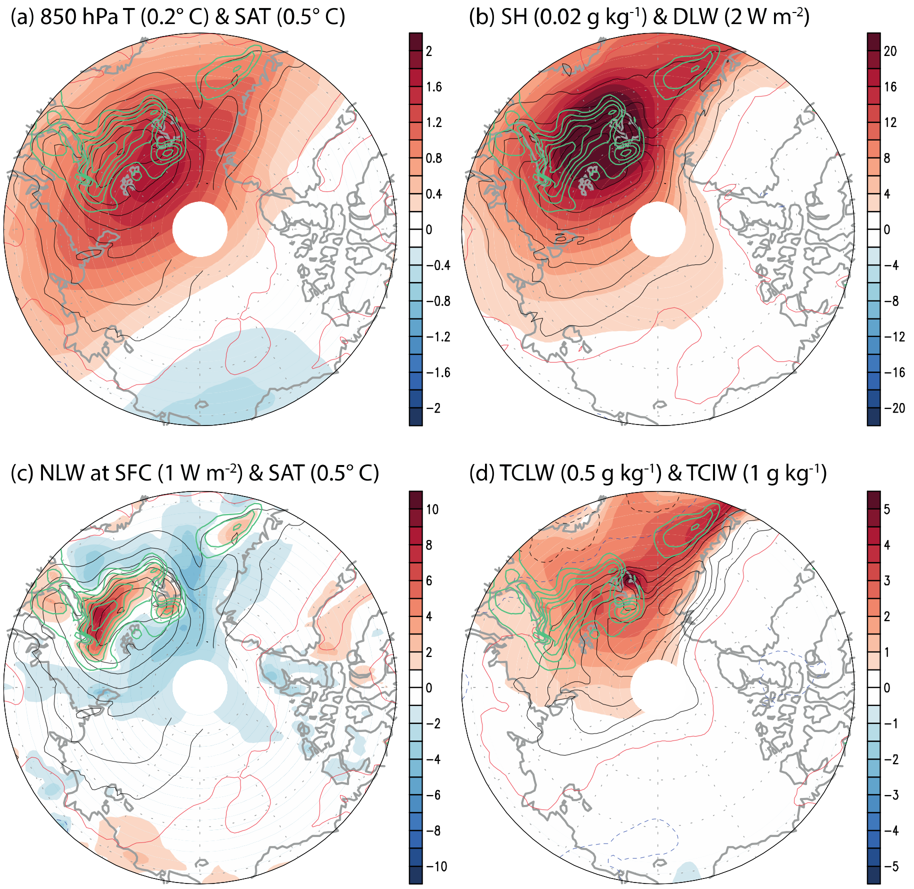

Figure S4. Winter-averaged patterns of (a) 850 hPa air temperature (*shading*) and 2 m air temperature (*contour*), (b) 900 hPa specific humidity (*shade*) and downward longwave radiation at surface (*contour*), (c) net (upward minus downward) longwave radiation at surface (*shade*) and SAT (*contour*), and (d) total cloud liquid water (*shade*) and total cloud ice water (*contour*) for the sea ice loss mode. The red contour is drawn at the value of the contour interval. The green contours in (a)–(d) represent the reduction of sea ice concentration. Figures were created with GrADS 2.1.0 (http://cola.gmu.edu/grads/).

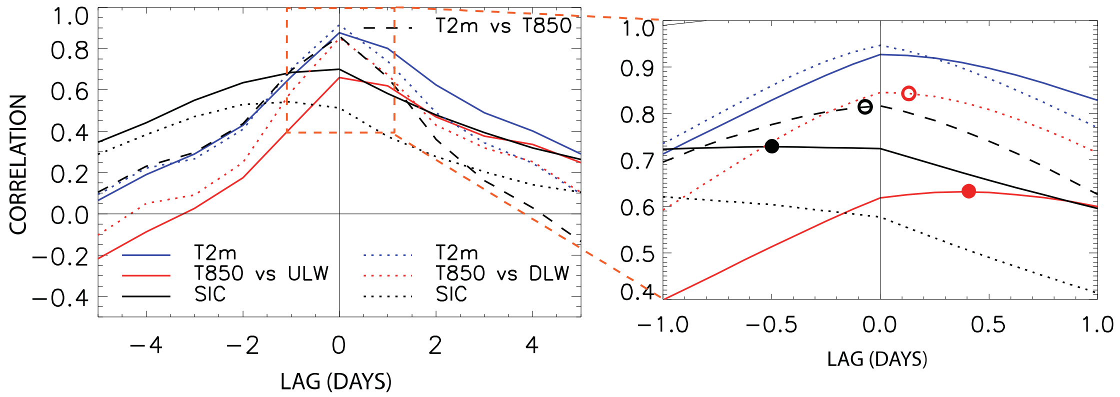

Figure S5. Lagged correlations: (a) correlation of upward (*solid lines*) and downward (*dotted lines*) longwave radiations with 2 m air temperature (*blue*), 850 hPa temperature (*red*), and sea ice concentration (*black*), and (b) a blowup of the boxed region in (a). Longwave radiation lags the other variable for a positive lag. Lagged correlation between 2 m air temperature and 850 hPa air temperature (*black dashed line*); 2 m air temperature leads 850 hPa temperature for a positive lag.

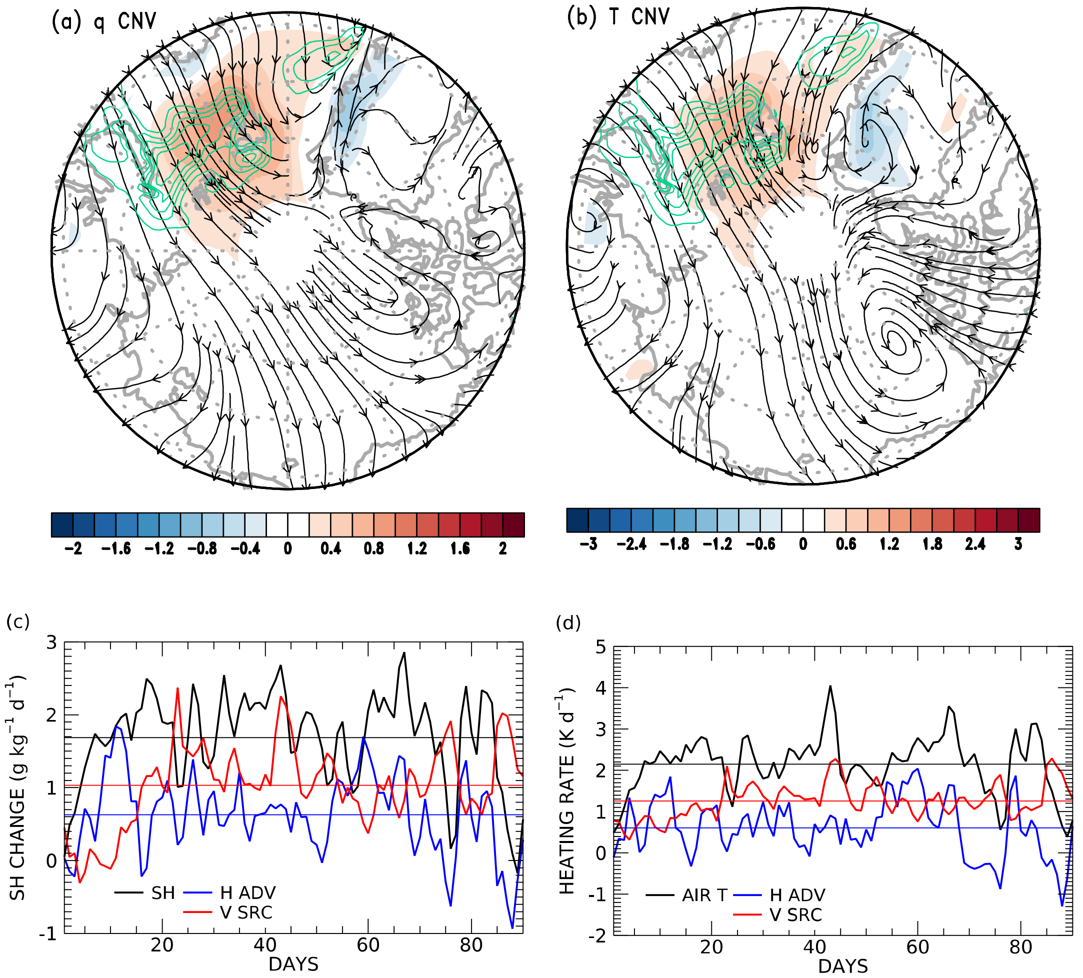

Figure S6. Winter-averaged (a) moisture transport (streamline) and its convergence (shade), and (b) heat transport (streamline) and its convergence (shade) in the lower troposphere (1000–850 hPa) associated with the sea ice loss mode. The green contours represent the reduction of sea ice concentration. Daily fluctuations of 1000–850 hPa averaged (c) specific humidity (SH), vertical source (V SRC; evaporation minus precipitation), and horizontal moisture transport (H ADV), (d) temperature (AIR T), the sum of turbulent flux and radiation (V SRC), and horizontal heat transport (H ADV). Figures in (a)–(b) were created with GrADS 2.1.0 (http://cola.gmu.edu/grads/).

As can be seen in Figure S6, there are net convergence of moisture transport and heat transport over the region of sea ice reduction, although the center of action is over the Greenland Sea. Thus, moisture and heat transport from lower latitudes apparently affects the variation of sea ice concentration, and it seems that both the convergence of moisture transport and the convergence of heat transport are at least partly responsible for the variation of specific humidity and

temperature in the lower troposphere. On the other hand, the convergence of horizontal transport of moisture and heat cannot explain one essential element of specific humidity and heat anomaly, respectively. Consider the following moisture and heat conservation equations:

$$\frac{\partial q}{\partial t} = -\vec{u} \cdot \nabla q + S = -\vec{u} \cdot \nabla_p q - \omega \frac{\partial q}{\partial p} + S, \tag{4}$$

$$\frac{\partial T}{\partial t} = -\vec{u} \cdot \nabla_p T - S_p \omega \doteq -\nabla_p \cdot \left(T\vec{u}\right) - S_p \omega = \frac{J}{c_p}. \tag{5}$$

We can compare the relative role of moisture and heat between horizontal convergence and vertical sources in the mechanism of Arctic amplification and sea ice loss. As can be seen in Fig. S6c, the net increase in specific humidity in the lower troposphere (1000–850 hPa), on average, is ~1.7 g kg^{-1} during winter due to sea ice loss. This amount is explained by addition of source term (~1 g kg^{-1}) and the convergence of horizontal moisture transport (~0.6 g kg^{-1}). Likewise, the sum of all the heating terms results in ~2.1 K increase in the atmospheric temperature in the lower troposphere (Fig. S6d). A little more than 1.2 K is explained by the turbulent heat flux plus the greenhouse effect due to increased specific humidity. The convergence of horizontal advection explains ~0.6 K increase in the lower tropospheric temperature.
